# Comparison of the Diagnostic Accuracies of Procalcitonin and C-Reactive Protein for Spontaneous Bacterial Peritonitis in Patients with Cirrhosis: A Systematic Review and Meta-Analysis

**DOI:** 10.3390/medicina61071134

**Published:** 2025-06-24

**Authors:** Tzu-Hsuan Tang, Ching-Min Lin, Kuang-Yu Niu, Shih-Hua Lin, Chen-Bin Chen, Chiao-Li Chuang, Chieh-Ching Yen

**Affiliations:** 1 Kaohsiung Chang Gung Memorial Hospital, Kaohsiung 833, Taiwan; violet890227@icloud.com; 2 Department of Emergency Medicine, Chang Gung Memorial Hospital, Linkou Branch, Taoyuan 333, Taiwan; arthurlin900045@gmail.com (C.-M.L.); peidra.niu@gmail.com (K.-Y.N.); 3 Department of Gastroenterology and Hepatology, New Taipei Municipal Tucheng Hospital, New Taipei City 236, Taiwan; 4 Department of Emergency Medicine, New Taipei Municipal Tucheng Hospital, New Taipei City 236, Taiwan; ans76ers@gmail.com; 5 Chang Gung Memorial Hospital, Linkou Branch, Taoyuan 333, Taiwan; julie0705046@gmail.com; 6 Institute of Emergency and Critical Care Medicine, National Yang Ming Chiao Tung University, Taipei 112, Taiwan; 7 College of Medicine, Chang Gung University, Taoyuan 333, Taiwan

**Keywords:** spontaneous bacterial peritonitis, biomarker, procalcitonin, C-reactive protein, meta-analysis

## Abstract

*Background and Objectives*: Spontaneous bacterial peritonitis (SBP) is both a prevalent and severe complication among individuals with cirrhosis. This systematic review and meta-analysis was designed to evaluate the diagnostic accuracy of procalcitonin (PCT) and compare it to C-reactive protein (CRP) in cirrhotic patients with suspected SBP. *Materials and Methods*: We performed an extensive literature review utilizing databases including MEDLINE, Embase, and the Cochrane Central Register of Controlled Trials. Original investigations reporting PCT diagnostic accuracy for SBP in cirrhotic populations were included. We computed pooled measures of sensitivity, specificity, positive and negative likelihood ratios, diagnostic odds ratio, and SROC curve area under the curve, with corresponding 95% confidence intervals (CIs). *Results*: Meta-analytical synthesis encompassed twenty eligible studies. Diagnostic accuracy analysis revealed PCT sensitivity of 0.73 (95% CI, 0.61–0.83) and specificity of 0.88 (95% CI, 0.83–0.91). Likelihood ratio yielded positive values of 6.0 (95% CI, 4.1–8.8) and negative values of 0.30 (95% CI, 0.20–0.47). Overall discriminative ability, quantified through SROC curve analysis, demonstrated an AUC of 0.90 (95% CI, 0.87–0.92). Head-to-head comparisons between PCT and CRP were available from ten studies, demonstrating PCT’s superior diagnostic accuracy over CRP, with significantly higher AUC values (PCT: 0.89, 95% CI 0.86–0.91; CRP: 0.74, 95% CI 0.70–0.78, *p* < 0.01). *Conclusions*: Although PCT demonstrates higher diagnostic accuracy than CRP, it does not appear to provide sufficient accuracy to support treatment decisions for SBP. We recommend not relying solely on the PCT test and advise that it be interpreted in conjunction with clinical findings.

## 1. Introduction

Cirrhosis compromises immune defenses, markedly increasing patients’ vulnerability to spontaneous bacterial infections, nosocomial infections, and infections by uncommon pathogens [1]. In cirrhotic individuals, such infections can provoke an exaggerated pro-inflammatory cytokine cascade that further destabilizes already fragile hemodynamics. Consequent deterioration may progress to septic shock, acute-on-chronic liver failure, multiple organ dysfunction, and death. Bacterial infections are a major concern among patients with cirrhosis, contributing to 30% to 50% of deaths in this group [2]. Among hospitalized patients with cirrhosis, the incidence of infections ranges from 32% to 34%, a rate four to five times higher than in the general hospitalized population [3]. Spontaneous bacterial peritonitis (SBP) is especially prevalent in cirrhotic patients with ascites, affecting 10% to 30% of those hospitalized. Approximately half of these SBP cases are present at admission, while the remainder develop during the hospital stay. The hospital mortality rate for SBP varies from 10% to 50%, influenced by various factors [4,5]. Diagnosing SBP involves analyzing the ascitic fluid cell count, typically performed using a microscope or automated cell counters, and conducting bacterial cultures [6]. However, performing paracentesis requires specialized skills and careful handling of specimens, which may not always be feasible in fast-paced clinical settings. Major complications such as bleeding and infection have been reported to occur at 1.6% [7]. Additionally, up to 60% of patients suspected of having SBP yield negative culture results, which complicates the diagnostic process [6]. Even patients with low polymorphonuclear (PMN) cell counts but positive bacterial cultures still require antibiotic treatment, highlighting the need for more effective diagnostic markers to improve SBP diagnosis.

Procalcitonin (PCT) and C-reactive protein (CRP) are widely used biomarkers for detecting inflammation and infection in clinical settings [8,9,10,11,12,13,14,15]. PCT, a precursor to calcitonin produced by thyroid C-cells, is usually at minimal levels in healthy individuals. Nevertheless, PCT concentrations demonstrate rapid elevation during bacterial infections, increasing within four hours following endotoxin exposure, reaching maximum levels at approximately eight hours, and sustaining elevation for up to 24 h [16]. Conversely, CRP represents an acute-phase protein produced by hepatic tissue in response to inflammatory processes, infections, or tissue damage. CRP concentrations begin increasing approximately six hours post-inflammatory stimulus, achieving peak levels around 36 h subsequently [17,18].

Current evidence regarding PCT’s effectiveness as a diagnostic tool for SBP stems from a 2015 meta-analysis that synthesized findings from seven studies [19]. However, this review did not include a comparison with CRP, and the limited number of studies included may compromise the reliability of the evidence. Given the significant increase in relevant research over the past decade, there is a need for an updated systematic review and meta-analysis.

## 2. Materials and Methods

We designed this systematic review to examine how effectively PCT measurements can diagnose SBP in the cirrhotic population. Our methodology incorporated comprehensive reporting standards, including PRISMA recommendations, Cochrane diagnostic test accuracy frameworks, and established review protocols [20,21,22]. The research process involved dual independent assessment by T.-H. T. and C.-M. L. for study inclusion, data synthesis, and methodological evaluation, while C.-C. Y. served as the arbitrating reviewer for consensus building. PROSPERO registration (CRD42024581722) was completed for the study protocol, and full PRISMA compliance documentation appears in Supplementary Table S1.

### 2.1. Data Sources and Searches

We executed a rigorous database search strategy targeting three major medical databases—MEDLINE (OvidSP), Embase, and CENTRAL—to capture diagnostic accuracy literature published before 29 August 2024. Our search framework combined controlled vocabulary (MeSH) with free-text terms addressing PCT, liver disease, cirrhosis, and SBP concepts, as documented in Supplementary Table S2. The search approach maintained broad inclusivity without temporal, geographic, or linguistic constraints. Manual screening of reference sections from candidate studies supplemented the electronic database searches.

### 2.2. Study Selection

The study selection process employed dual independent review by T.-H. T. and C.-M. L., beginning with title and abstract screening of the retrieved literature to identify potentially relevant studies. Full-text assessment followed for articles meeting preliminary criteria, with C.-C. Y. resolving reviewer disagreements through consensus discussion. Our inclusion framework targeted diagnostic accuracy studies (both prospective and retrospective design) examining PCT’s role in SBP detection among hospitalized adults (minimum age 18 years) with liver disease. We systematically excluded case studies, conference materials, animal experiments, reviews, and research with participant overlap, prioritizing the most comprehensive study when database duplication occurred. Essential data requirements included sufficient information for constructing diagnostic accuracy tables (true/false positives and negatives), obtainable either from published results or calculated using sensitivity/specificity values. Missing data prompted direct author correspondence for clarification. Quality assurance involved testing inclusion criteria reliability on 10% of randomly selected articles, with Cohen’s kappa measuring reviewer concordance.

### 2.3. Data Extraction and Quality Assessment

Data synthesis and quality assessment were systematically performed by the two reviewers using predetermined extraction protocols, with C.-C. Y. providing arbitration when consensus could not be reached. The comprehensive data collection captured study-specific details such as research setting, enrollment parameters, participant characteristics, biomarker cutoff thresholds (PCT and CRP), and established SBP diagnostic protocols. Diagnostic performance metrics were systematically documented, including the four-cell contingency table data and test accuracy measures (sensitivity/specificity) for both PCT and CRP where available. We applied the QUADAS-2 framework to assess methodological rigor and external validity, examining potential bias across four domains: patient selection, index test, reference standard, and flow and timing [23].

### 2.4. Data Synthesis and Analysis

Individual study diagnostic performance was determined by constructing 2 × 2 diagnostic tables from available data. Studies reporting multiple cutoff values underwent selection based on either pre-established thresholds or, when unspecified, values optimizing the Youden index (J = Sensitivity − (1 − Specificity)). Our analytical strategy employed bivariate modeling that accounts for both fixed and random threshold and accuracy effects, enabling comprehensive estimation of diagnostic metrics including sensitivity, specificity, likelihood ratios (positive and negative), and diagnostic odds ratios [24]. We further developed hierarchical summary ROC (HSROC) models to create summary curves illustrating sensitivity against 1-specificity relationships [25,26]. Visual representation included 95% confidence and prediction ellipses around pooled data to convey estimate reliability and between-study heterogeneity. Heterogeneity assessment involved graphical examination of forest plots and ROC space patterns. Planned subgroup investigations targeted standard PCT cutoffs (0.5–1 ng/mL) and studies with exclusively cirrhotic participants, removing mixed liver disease populations. We conducted two sensitivity analyses to test result stability: excluding case–control studies and studies using optimal cutoff. Deeks’ funnel plot assessed publication bias by plotting effective sample size against log diagnostic odds ratios. Evidence synthesis incorporated 95% confidence intervals for all parameters. We tabulated PCT diagnostic effectiveness findings and applied GRADE criteria to evaluate evidence quality and confidence levels [27,28]. All analyses were performed using STATA version 17, utilizing Metadta for pooled estimates and SROC curves, and Midas for publication bias assessment.

## 3. Results

### 3.1. Search Results

**Figure 1:**
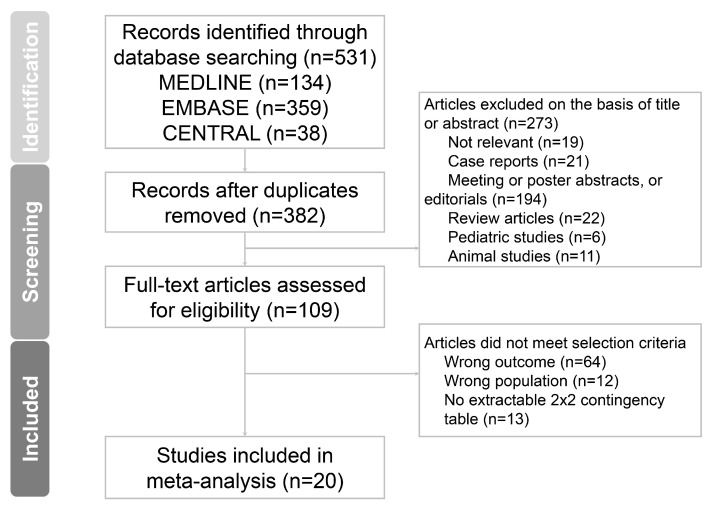
Flow chart of study identification, screening, inclusion, and exclusion for meta-analysis.

The database search identified 531 publications. Following preliminary screening, 422 publications were eliminated, with 109 remaining for comprehensive full-text evaluation. Subsequently, 89 additional articles were excluded, culminating in 20 studies selected for final analysis (Figure 1) [29,30,31,32,33,34,35,36,37,38,39,40,41,42,43,44,45,46,47,48]. Manual review of reference sections from these selected articles failed to identify any further qualifying research. Inter-reviewer concordance for study selection reached 91.8%, achieving a Cohen’s kappa coefficient of k = 0.76.

### 3.2. Study Characteristics

Study characteristics are detailed in Supplementary Table S3. The included investigations spanned publication years 2000–2023, with a median participant count of 82 (interquartile range: 50–199), totaling 6044 patients across all analyses. Regional distribution showed European studies comprising 5 investigations (25%) [37,38,43,47,48], Asian studies representing 10 investigations (50%) [29,30,33,36,39,41,42,44,45,46], African studies accounting for 4 investigations (20%) [32,34,35,40], and North American studies contributing 1 investigation (5%) [31]. Regarding the study design, 10 studies (50%) were prospective cohort studies [31,33,34,35,36,37,38,40,43,47], 6 (30%) were retrospective cohort studies [29,30,39,42,44,46], 1 (5%) was a case–control study [41], and 3 (15%) did not report the design. Of the total 6044 participants, 1780 individuals (29.5%) comprised the SBP cohort, while 4264 participants (70.5%) formed the control cohort. SBP prevalence across individual studies varied between 18% and 65.8%. PCT threshold values for infection detection spanned from 0.35 (ng/mL) to 2 (ng/mL), with a median threshold of 0.67 (ng/mL). Sixteen (80%) studies included 100% cirrhotic patients [30,31,32,33,34,35,36,37,38,39,40,41,43,45,47,48], while four (20%) included a mixed population [29,42,44,46]. SBP in the included studies was defined as the presence of more than 250 PMN cells/mm^3^ in an ascites sample, with or without a positive culture, combined with clinically compatible symptoms.

### 3.3. Quality Assessment

QUADAS-2 evaluation results are presented in Supplementary Table S4 and Supplementary Figure S1. Within the domain of patient selection, two studies (10%) were identified as having a high risk of bias, one due to its case–control design and another due to non-consecutive patient enrollment [29,41]. Regarding the index test, 13 studies (65%) were noted for high risk of bias, as they used optimal cutoff values rather than predefined ones to calculate sensitivity and specificity [29,32,33,34,35,36,38,39,40,42,44,46,48]. In the flow and timing domain, three studies (15%) showed an unclear risk of bias due to undocumented blood sample collection times [30,32,35]. Concerning applicability, the patient selection domain in four studies (20%) had an unclear risk of bias because they involved specific populations [35,40,44,46].

### 3.4. Primary Analysis of Overall Accuracy

**Figure 2:**
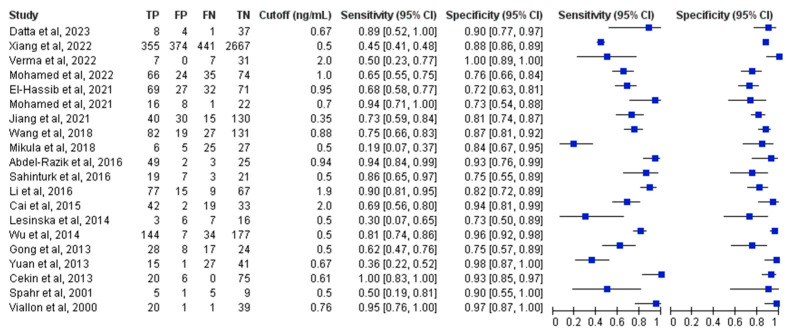
Forest plots of the sensitivity and specificity for procalcitonin across all included studies with varying cutoffs. TP: true positive; FP: false positive; FN: false negative; TN: true negative; CI: confidence interval [29,30,31,32,33,34,35,36,37,38,39,40,41,42,43,44,45,46,47,48].

**Figure 3:**
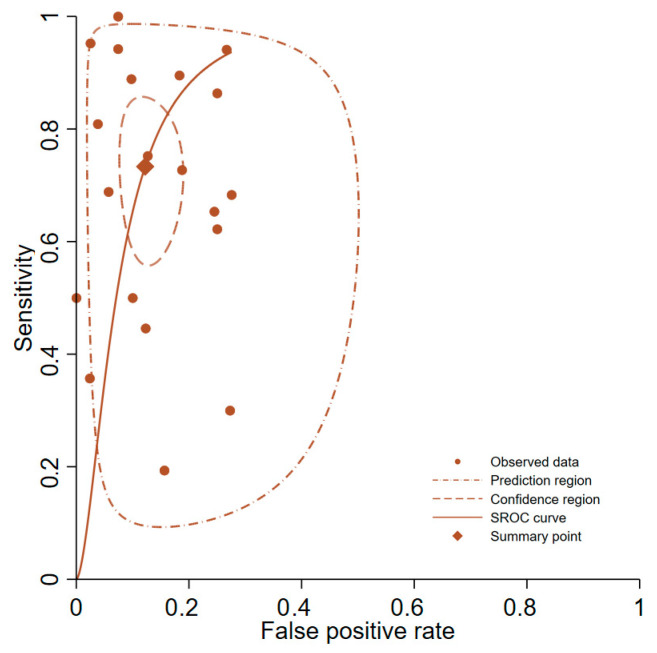
SROC curves for the diagnostic accuracy of procalcitonin for spontaneous bacterial peritonitis. SROC: summary receiver operating characteristic.

Forest plot representations of PCT sensitivity and specificity from the 20 analyzed studies are displayed in Figure 2. Pooled sensitivity across all investigations reached 0.73 (95% CI, 0.61–0.83), while pooled specificity achieved 0.88 (95% CI, 0.83–0.91). The pooled diagnostic odds ratio measured 19.7 (95% CI, 9.6–40.5). Pooled positive and negative likelihood ratio estimates were 6.0 (95% CI, 4.1–8.8) and 0.30 (95% CI, 0.20–0.47), respectively. Figure 3 presents the SROC curves alongside bivariate summary points for specificity and sensitivity with corresponding 95% confidence regions. The SROC curve area under the curve (AUC) measured 0.90 (95% CI, 0.87–0.92). Post-test probability calculations for both ‘positive’ and ‘negative’ PCT results were determined using summary estimates across varying pretest probabilities in patients evaluated for suspected SBP through PCT testing (Table 1).

### 3.5. Subgroup and Sensitivity Analyses

Considerable heterogeneity was evident among the analyzed studies, as demonstrated by the wide variation in sensitivity and specificity values. We performed subgroup analyses to investigate possible causes of this heterogeneity (Table 2). Sixteen studies used a cutoff of 0.5–1 ng/mL and demonstrated similar summary estimates (Supplementary Figure S2). Sixteen studies included 100% cirrhotic patients and showed a significantly lower specificity (0.85; 95% CI, 0.82–0.91) compared to the original model without this covariate-by-likelihood ratio test (*p* = 0.02) (Supplementary Figure S3). Sensitivity analyses demonstrated that studies excluding case–control design (*n* = 19) had similar summary estimates (Supplementary Figure S4). However, studies excluding those using optimal cutoffs (*n* = 8) showed a significantly lower sensitivity (0.52; 95% CI 0.37–0.67) compared to the original model without this covariate-by-likelihood ratio test (*p* = 0.02) (Supplementary Figure S5).

### 3.6. Head-to-Head Performance Analysis of PCT and CRP

**Figure 4:**
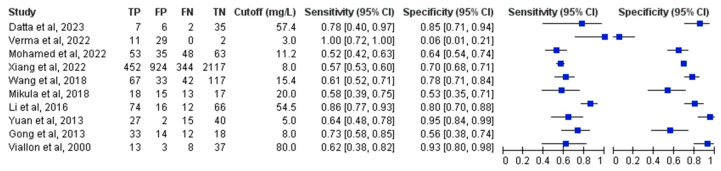
Forest plots of the sensitivity and specificity for C-reactive protein across all included studies with varying cutoffs. TP: true positive; FP: false positive; FN: false negative; TN: true negative; CI: confidence interval [29,30,31,32,36,37,39,44,45,48].

**Figure 5:**
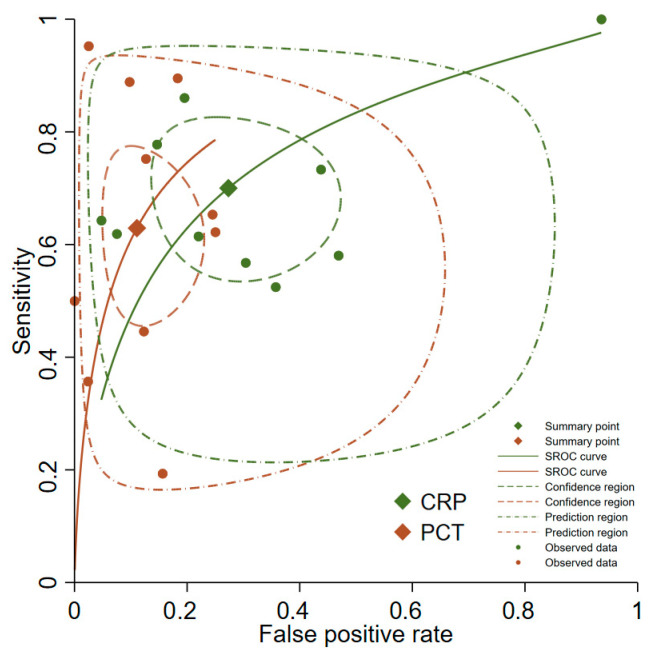
Comparisons of procalcitonin and C-reactive protein with SROC curves and 95% confidence interval region. PCT: procalcitonin; CRP: C-reactive protein; SROC: summary receiver operating characteristic.

Direct biomarker comparisons were available from 10 of the included 20 studies (Figure 4) [29,30,31,32,36,37,39,44,45,48]. The pooled PCT sensitivity and specificity measured 0.63 (95% CI, 0.52–0.73) and 0.89 (95% CI, 0.82–0.93), while CRP values reached 0.70 (95% CI, 0.59–0.79) and 0.73 (95% CI, 0.60–0.82), respectively. PCT demonstrated significantly superior diagnostic accuracy compared to CRP when assessed by AUC (0.89; 95% CI 0.86–0.91 versus 0.74; 95% CI, 0.70–0.78, *p* < 0.01) (Figure 5).

### 3.7. Publication Bias

Publication bias was statistically significant among the 20 PCT-related studies (*p* = 0.02), whereas the 10 CRP-related studies demonstrated no statistically significant publication bias (*p* = 0.14) (Supplementary Figure S6).

### 3.8. Certainty of Evidence

PCT certainty of evidence received ratings of ‘very low’ for sensitivity and ‘low’ for specificity, attributed to bias risks, inconsistency, and publication bias concerns. In contrast, CRP certainty of evidence achieved ‘moderate’ ratings for both sensitivity and specificity, with bias risk as the primary limiting factor. Comprehensive GRADE evidence profiles documenting these assessments appear in Supplementary Table S5.

## 4. Discussion

This systematic review and meta-analysis represents the most comprehensive evaluation of PCT and CRP diagnostic accuracy for SBP to date. Analysis of 20 investigations encompassing 6044 participants demonstrated PCT’s potential superiority over CRP in SBP diagnosis, evidenced by higher pooled AUC values (PCT: 0.89 versus CRP: 0.74 for direct comparison). Despite achieving moderate-to-high diagnostic accuracy measured by AUC, our findings suggest PCT’s clinical utility for initial triage of cirrhotic patients with suspected SBP remains constrained. Applying pooled diagnostic parameters (sensitivity: 0.73, specificity: 0.88) to a theoretical population of 1000 patients with 50% SBP prevalence would generate 135 undetected SBP cases (false-negatives) and 60 incorrectly diagnosed cases (false-positives). For patients presenting with 50% pretest SBP probability, PCT incorporation would modify post-test probabilities to 22% following negative results (insufficient for SBP exclusion) and 88% following positive results (inadequate for definitive SBP confirmation). Optimal biomarker utilization in SBP detection requires weighing missed diagnosis risks against unnecessary antibiotic treatment benefits, considering local SBP prevalence patterns. Given SBP’s significant mortality burden, identifying highly sensitive biomarkers for prompt detection remains paramount. Considering suboptimal sensitivity and negative predictive value, we recommend against PCT alone for SBP screening in suspected cases.

The varied performance of PCT in diagnosing infections in liver diseases may be attributed to multiple factors. While it is theoretically expected that liver diseases might suppress PCT synthesis, clinical findings reveal a more complex reality. For example, patients with steatohepatitis and simple steatosis exhibit serum PCT levels similar to those of healthy control groups [49]. Conversely, cirrhotic individuals commonly demonstrate raised PCT baseline levels independent of bacterial processes, with this elevation occurring across all disease etiologies and severity grades [50]. Multiple forms of acute hepatic insult—including alcohol-mediated injury, viral hepatitis, and progressive fibrotic changes—can drive PCT levels beyond the 0.5 ng/mL threshold through non-infectious mechanisms [51]. Research by Sugihara et al. confirmed this phenomenon in acute liver failure populations, where PCT elevation occurred relative to non-ALF controls despite the absence of confirmed bacterial infections [52]. The mechanisms underlying these elevated PCT levels remain unclear, but two possible explanations have been proposed: bacterial translocation leading to endotoxemia and systemic inflammatory response syndrome, or a pro-inflammatory cytokine response that could create a positive feedback loop, further increasing PCT levels [53,54,55,56,57]. Such evidence reveals the multifaceted interaction between liver dysfunction and PCT elevation patterns.

The methodological rigor of our investigation includes multiple key advantages: unrestricted comprehensive database searching across all languages, robust analytical approaches through subgroup and sensitivity testing, and stringent quality evaluation protocols. We used a direct comparison approach, testing all patients with both PCT and CRP to evaluate their diagnostic accuracies for SBP. This Cochrane-recommended method ensures that each study serves as its own control, reducing confounding factors [20]. Yang et al. conducted a similar meta-analysis in 2015, which concluded that PCT may accurately diagnose SBP in end-stage liver disease [19]. However, the analysis is limited by only seven studies, and the conclusion is not entirely supported by a sensitivity of 0.82, corresponding to 18% of patients not receiving adequate treatment, and a specificity of 0.86, corresponding to 14% being unnecessarily treated.

Between-study variability in diagnostic accuracy parameters was apparent through forest plot visualization, consistent with typical patterns observed across diagnostic test accuracy meta-analyses [58,59,60]. Differences in the inclusion and exclusion criteria may account for a large portion of the heterogeneity. Our subgroup analyses showed that studies with 100% cirrhotic patients have a significantly lower specificity compared to the original model without this covariate. This reflects the diagnostic uncertainty of PCT in cirrhosis. Variability across studies regarding clinical criteria for SBP evaluation likely constitutes an additional important source of heterogeneity. We analyzed studies with common cutoffs between 0.5 and 1 ng/mL and found similar summary estimates. Additional elements potentially influencing the noted heterogeneity encompass assay sensitivity variations, methodological quality differences, disease severity disparities, population diversity, and SBP prevalence fluctuations. Nevertheless, limited reporting details and restricted study numbers prevented exploration of these factors’ potential relationships. Various biases were present throughout the included investigation. One of the main biases is that several studies used the optimal cutoffs post hoc based on the Youden index, rather than using predefined ones. Our sensitivity analysis involved removing investigations that employed optimal cutoffs, revealing that studies utilizing predetermined thresholds exhibited significantly reduced sensitivity relative to the initial model lacking this variable. This finding may result from the inflated performance associated with optimal cutoff selection.

Our review has several limitations. First, although we performed subgroup and sensitivity evaluations, the diversity across included investigations highlights heterogeneity in liver disease etiologies and severity levels, restricting our findings’ external validity. Second, examining lower PCT thresholds would enhance screening clinical applicability, but this exploration proved impossible since only one investigation employed cutoffs under 0.5 ng/mL. Third, multiple studies demonstrated elevated bias risk across one or more QUADAS-2 domains, potentially compromising evidence quality. Finally, our serum PCT meta-analysis exhibited substantial publication bias. Such bias frequently occurs in diagnostic research and may pose greater difficulties than in randomized controlled studies [61,62]. Studies yielding unfavorable outcomes were potentially less likely to achieve publication, possibly artificially elevating perceived test diagnostic performance within the meta-analysis.

## 5. Conclusions

This systematic review and meta-analysis demonstrates that among 6044 patients from 20 studies, PCT has moderate-to-high diagnostic accuracy, while CRP has low accuracy for SBP in cirrhotic patients. Timely diagnosis of SBP remains an important goal in clinical practice. However, given the suboptimal sensitivity and negative predictive value, relying solely on PCT does not seem to provide sufficient accuracy to support treatment decisions. Instead, both biomarkers should complement, not replace, thorough clinical evaluations. Future research efforts might focus on strategies such as combining various biomarkers to construct a diagnostic panel or clinical score, treatment regimen of serial biomarker measurement, and exploring newer molecular markers to improve diagnostic accuracy for SBP.

## Figures and Tables

**Table 1:** Post-test probabilities for spontaneous bacterial peritonitis for a sample of population prevalence, determined using procalcitonin.

Pretest Probability	Post-test Probability After a Positive Result	Post-test Probability After a Negative Result	False Positive *	False Negative *
0.1	0.44	0.03	108	27
0.2	0.64	0.07	96	54
0.3	0.75	0.11	84	81
0.4	0.82	0.16	72	108
0.5	0.88	0.22	60	135
0.6	0.91	0.30	48	162
0.7	0.94	0.40	36	189

* Number of false positives and negatives in 1000 hypothetical cases.

**Table 2:** Summary of subgroup and sensitivity analyses of procalcitonin in the diagnosis of spontaneous bacterial peritonitis.

Subgroup	Number of Studies	Pooled Sensitivity (95% CI)	SUBGROUP *p* Value in Sensitivity	Pooled Specificity (95% CI)	SUBGROUP *p* Value in Specificity	Positive Likelihood Ratio	Negative Likelihood Ratio	Pooled AUC (95% CI)	Diagnostic Odds Ratio
Overall group	20	0.73 (0.61–0.83)	-	0.88 (0.83–0.91)	-	6.0 (4.1–8.8)	0.30 (0.20–0.47)	0.90 (0.87–0.92)	19.7 (9.6–40.5)
PCT cutoff 0.5–1 (ng/mL)	16	0.74 (0.58–0.85)	0.87	0.87 (0.82–0.91)	0.71	5.8 (3.7–9.0)	0.30 (0.17–0.52)	0.89 (0.86–0.92)	19.2 (7.7–47.9)
100% cirrhotic patients	16	0.71 (0.58–0.81)	0.35	0.85 (0.80–0.88)	0.01 *	4.6 (3.3–6.5)	0.34 (0.23–0.52)	0.87 (0.84–0.89)	13.4 (6.7–26.8)
Excluding case–control study	19	0.74 (0.60–0.84)	0.83	0.87 (0.82–0.91)	0.42	5.8 (3.9–8.6)	0.30 (0.19–0.48)	0.90 (0.87–0.93)	19.4 (9.1–41.5)
Excluding studies using optimal cutoffs	8	0.52 (0.37–0.67)	0.01 *	0.86 (0.79–0.91)	0.57	3.8 (2.3–6.5)	0.55 (0.40–0.77)	0.82 (0.78–0.85)	6.9 (3.1–15.3)

PCT: procalcitonin; CI: confidence interval; AUC: area under the curve. * *p* < 0.05.

## Data Availability

Our investigation relies on synthesized findings from cited studies, with source data accessible through the primary manuscripts.

## Supplementary Materials

The following supporting information can be downloaded at: https://www.mdpi.com/article/10.3390/medicina61071134/s1, Figure S1: Quality assessment for 44 studies (QUADAS-2); Figure S2: (A) SROC curves and (B) forest plots for the diagnostic accuracy of procalcitonin for spontaneous bacterial peritonitis in studies using a cutoff of 0.5–1 ng/mL.; Figure S3: (A) SROC curves and (B) forest plots for the diagnostic accuracy of procalcitonin for spontaneous bacterial peritonitis in studies including 100% cirrhotic patients.; Figure S4: (A) SROC curves and (B) forest plots for the diagnostic accuracy of procalcitonin for spontaneous bacterial peritonitis in studies excluding case-control design.; Figure S5: (A) SROC curves and (B) forest plots for the diagnostic accuracy of procalcitonin for spontaneous bacterial peritonitis in studies excluding those using optimal cutoffs.; Figure S6: Deeks’ funnel plot (asymmetry test) for (A) PCT and (B) CRP.; Table S1: PRISMA checklist; Table S2: Electronic search strategies; Table S3: Main characteristics of the included studies; Table S4: Risk of bias and concerns of applicability for the 20 studies included in this review; Table S5: GRADE evidence profile to determine the diagnostic accuracy of procalcitonin and C-reactive protein.

## Abbreviations

The following abbreviations are used in this manuscript:

PCT	procalcitonin
SBP	spontaneous bacterial peritonitis
CRP	C-reactive protein
SROC	summary receiver operating characteristic
HSROC	hierarchical summary receiver operating characteristic
