# Supplementary material for: Comparison of the Diagnostic Accuracies of Procalcitonin and C-Reactive Protein for Spontaneous Bacterial Peritonitis in Patients with Cirrhosis: A Systematic Review and Meta-Analysis
Source: Medicina (Kaunas). 2025 Jun 24;61(7):1134. doi: 10.3390/medicina61071134 (PMC12298292; doi:10.3390/medicina61071134)

Supplementary Table S1. PRISMA checklist

Section/topic	#	PRISMA-DTA Checklist Item	Reported on page #
**TITLE / ABSTRACT**			
Title	1	Identify the report as a systematic review (+/- meta-analysis) of diagnostic test accuracy (DTA) studies.	1
Abstract	2	Abstract: See PRISMA-DTA for abstracts.	1
**INTRODUCTION**			
Rationale	3	Describe the rationale for the review in the context of what is already known.	2
Clinical role of index test	D1	State the scientific and clinical background, including the intended use and clinical role of the index test, and if applicable, the rationale for minimally acceptable test accuracy (or minimum difference in accuracy for comparative design).	2
Objectives	4	Provide an explicit statement of question(s) being addressed in terms of participants, index test(s), and target condition(s).	2
**METHODS**			
Protocol and registration	5	Indicate if a review protocol exists, if and where it can be accessed (e.g., Web address), and, if available, provide registration information including registration number.	2
Eligibility criteria	6	Specify study characteristics (participants, setting, index test(s), reference standard(s), target condition(s), and study design) and report characteristics (e.g., years considered, language, publication status) used as criteria for eligibility, giving rationale.	3
Information sources	7	Describe all information sources (e.g., databases with dates of coverage, contact with study authors to identify additional studies) in the search and date last searched.	3
Search	8	Present full search strategies for all electronic databases and other sources searched, including any limits used, such that they could be repeated.	3
Study selection	9	State the process for selecting studies (i.e., screening, eligibility, included in systematic review, and, if applicable,	3

		included in the meta-analysis).	
Data collection process	10	Describe method of data extraction from reports (e.g., piloted forms, independently, in duplicate) and any processes for obtaining and confirming data from investigators.	3
Definitions for data extraction	11	Provide definitions used in data extraction and classifications of target condition(s), index test(s), reference standard(s) and other characteristics (e.g. study design, clinical setting).	3
Risk of bias and applicability	12	Describe methods used for assessing risk of bias in individual studies and concerns regarding the applicability to the review question.	3
Diagnostic accuracy measures	13	State the principal diagnostic accuracy measure(s) reported (e.g. sensitivity, specificity) and state the unit of assessment (e.g. per-patient, per-lesion).	3
Synthesis of results	14	Describe methods of handling data, combining results of studies and describing variability between studies. This could include, but is not limited to: a) handling of multiple definitions of target condition. b) handling of multiple thresholds of test positivity, c) handling multiple index test readers, d) handling of indeterminate test results, e) grouping and comparing tests, f) handling of different reference standards	3
Meta-analysis	D2	Report the statistical methods used for meta-analyses, if performed.	4
Additional analyses	16	Describe methods of additional analyses (e.g., sensitivity or subgroup analyses, meta-regression), if done, indicating which were pre-specified.	4
**RESULTS**			
Study selection	17	Provide numbers of studies screened, assessed for eligibility, included in the review (and included in meta-analysis, if applicable) with reasons for exclusions at each stage, ideally with a flow diagram.	5
Study characteristics	18	For each included study provide citations and present key characteristics including: a) participant characteristics (presentation, prior testing), b) clinical setting, c) study	5

		design, d) target condition definition, e) index test, f) reference standard, g) sample size, h) funding sources	
Risk of bias and applicability	19	Present evaluation of risk of bias and concerns regarding applicability for each study.	14
Results of individual studies	20	For each analysis in each study (e.g. unique combination of index test, reference standard, and positivity threshold) report 2x2 data (TP, FP, FN, TN) with estimates of diagnostic accuracy and confidence intervals, ideally with a forest or receiver operator characteristic (ROC) plot.	14
Synthesis of results	21	Describe test accuracy, including variability; if meta-analysis was done, include results and confidence intervals.	14
Additional analysis	23	Give results of additional analyses, if done (e.g., sensitivity or subgroup analyses, meta-regression; analysis of index test: failure rates, proportion of inconclusive results, adverse events).	15
**DISCUSSION**			
Summary of evidence	24	Summarize the main findings including the strength of evidence.	16
Limitations	25	Discuss limitations from included studies (e.g. risk of bias and concerns regarding applicability) and from the review process (e.g. incomplete retrieval of identified research).	17
Conclusions	26	Provide a general interpretation of the results in the context of other evidence. Discuss implications for future research and clinical practice (e.g. the intended use and clinical role of the index test).	17
**FUNDING**			
Funding	27	For the systematic review, describe the sources of funding and other support and the role of the funders.	18

Supplementary Table S2. Electronic search strategies

(A) MEDLINE (OvidSP)

Date limits: inception to August 29, 2024

	Search strategy	Results
1	liver disease.mp.	124,421
2	cirrhosis.mp.	156,193
3	hepatitis.mp.	288,986
4	(procalcitonin or pro-calcitonin).mp.	10,140
5	Infections/	40,878
6	Infection$.mp.	2,425,711
7	cirrhotic.mp.	25,542
8	peritonitis.mp.	45,037
9	bacter$.mp.	1,848,787
10	1 or 2 or 3 or 7	468,323
11	5 or 6 or 7 or 8 or 9	3,715,297
12	4 and 10 and 11	134

(B) Embase

Date limits: inception to August 29, 2024

	Search strategy	Results
1	'liver disease':ti,ab	186,951
2	'cirrhosis':ti,ab	179,185
3	'cirrhotic':ti,ab	46,179
4	'hepatitis':ti,ab	361,321
5	'procalcitonin':ti,ab OR 'pro-calcitonin':ti,ab	16,608
6	'infections'/de	346,681
7	infection*:ti,ab	2,370,944
8	peritonitis:ti,ab	48,539
9	bacter*:ti,ab	1,287,426
10	#1 OR #2 OR #3 OR #4	602,217
11	#6 OR #7 OR #8 OR #9	3,423,620
12	#5 AND #10 AND #11	359

(C) CENTRAL

Date limits: inception to August 29, 2024

	Search strategy	Results
1	liver disease	33,559

2	cirrhosis	12,230
3	cirrhotic	3,558
4	hepatitis	23,931
5	procalcitonin	1577
6	infections	82,923
7	peritonitis	2,792
8	bacter*	56,795
9	1 OR 2 OR 3 OR 4	58,145
10	6 OR 7 OR 8	118,441
11	5 AND 9 AND 10	38

Supplementary Table S3. Main characteristics of the included studies

Author	Year	Country	Study design	Setting	Sample size	Prevalence (SBP)	Mean age, years[†]	Biomarkers studied	Population	Diagnostic crite[illegible] for SBP
Datta	2023	Bangladesh	RS	Hospital	50	18.0%	57.8	PCT, CRP	Decompensated chronic liver disease	Ascitic PMN [illegible] 250/mm³
Xiang	2022	China	RS	Hospital	3837	20.7%	53 (median)	PCT, CRP	Decompensated cirrhosis	Two of the following: clini[illegible] signs, ascitic P[illegible] ≥ 250/mm³, o[illegible] positive bacter[illegible] culture of ascit[illegible]
Verma	2022	USA	PS	Hospital	45	31.1%	53.8	PCT, CRP	Cirrhosis	Ascites WBC [illegible] 500/mm³ and P[illegible] > 250/mm³ wi[illegible] positive ascite[illegible] bacterial cultu[illegible]
Mohamed	2022	Egypt	NA	Ward	199	50.8%	55.1	PCT, CRP, MCP-1	Decompensated cirrhosis	Ascitic PMN [illegible] 250/mm³

Mohamed	2021	Egypt	PS	Hospital	47	36.2%	63.1	WBC/PLT, calprotectin	Decompensated HCV cirrhosis	Clinical signs or ascitic PMN ≥ 250/mm³	CTP B (66%), CTP C (34%)	NA	NA
Jiang	2021	China	PS	Ward	215	25.6%	40.1	PCT, PMN	Cirrhosis	NA	CTP A (3.3%), CTP B (24.7%), CTP C (72.1%)	NA	Prior to treatment
El-Hassib	2021	Egypt	PS	Ward	199	50.8%	57.5	PCT, MicroRNA	Cirrhosis	NA	CTP B (34.7%), CTP C (67.8%)	ELISA	During paracentesis of ascitic fluid
Wang	2018	China	PS	Ward	259	42.1%	51.4	PCT, CRP, sNFI, dCHC	Cirrhosis	Ascitic PMN ≥ 250/mm³	Child B (40.9%), Child C (59.1%)	Electrochemiluminescence immunoassay method, measured using an immunology analyzer (Cobas E601, Roche Diagnostics)	On admission
Mikuła	2018	Poland	PS	Ward	63	49.2%	61.3	PCT, CRP, D-dimer, HBP	Decompensated cirrhosis	Ascitic PMN ≥ 250/mm³	NA	NA	Within 24 hours of admission
Şahintürk	2016	Turkey	PS	Ward	50	44.0%	67.08	PCT	Cirrhosis	Ascitic PMN ≥ 250/mm³	CTP A (16%), CTP B (48%),	Cobas immunoassay analyzer (Roche	During paracentesis

											CTP C (36%)	Diagnostics) using electrochemiluminescence immunoassay method (Brahms Diagnostica, Berlin, Germany)	
Li	2016	China	RS	Hospital	168	51.2%	54.5	PCT, CRP	Cirrhosis	Clinical signs and ascitic PMN ≥ 250/mm³	CTP B (9.3%), CTP C (90.7%)	NA	On admission
Abdel-Razik	2016	Egypt	PS	Ward	79	65.8%	58.1	PCT, PMN, calprotectin, TNF-α, IL-6	Cirrhosis with HCC	Ascitic PMN ≥ 250/mm³	CTP B (25%), CTP C (75%)	RayBio® Human PCT ELISA kit (RayBiotech Inc., Norcross, GA, USA)	At paracentesis
Cai	2015	China	Case-control	Hospital	96	63.5%	59.3	PCT, WBC/PLT	Decompensated cirrhosis	Ascitic PMN ≥ 250/mm³	CTP B (5.3%), CTP C (94.7%)	Automated enzyme-linked fluorescence quantitative analyzer (VIDAS, Merieux, France)	At admission before antibiotics
Wu	2014	China	RS	Hospital	362	49.2%	50.8	PCT	Cirrhosis and hepatitis	Clinical signs or PMN count ≥250 cells/mm³ with positive culture	CTP A (8.1%), CTP B (36.5%), CTP C	Electrochemiluminescence assay (ECLIA) on a Roche Elecsys analyzer	On admission

											(55.4%)		
Lesińska	2014	Poland	PS	Ward	32	31.3%	49.5	MIP-1β	Decompensated cirrhosis	Ascitic PMN ≥ 250/mm³	CTP A (3.1%), CTP B (46.9%), CTP C (50%).	LUMItest PCT (B.R.A.H.M.S.- Diagnostica, Berlin, Germany)	First day of admission
Yuan	2013	China	RS	Hospital	84	50.0%	55.4	PCT, CRP	Chronic severe hepatitis B	Ascitic PMN ≥ 250/mm³	NA	Liaison analyzer (Diasorin, Saluggia, Italy)	On admission
Gong	2013	China	NA	Ward	77	58.4%	37.8	CRP, HNP	Cirrhosis	Clinical signs with positive ascites culture or ascitic PMN ≥ 250/mm³	NA	LUMItest PCT (B.R.A.H.M.S.- Diagnostica, Berlin, Germany)	Day of or after SBP diagnosis
Cekin	2013	Turkey	RS	Ward	101	19.8%	63.4	PCT	Cirrhosis and malignancy-related ascites	Ascites WBC ≥ 500/mm³ and PMN > 250/mm³ with positive ascites bacterial culture	NA	Cobas immunoassay (Roche Diagnostics)	Simultaneously with ascitic fluid samples
Spahr	2001	Switzerland	PS	Hospital	20	50.0%	58.0	PCT, IL-6	Cirrhosis	Clinical signs and ascitic PMN ≥ 250/mm³	CTP B (50%), CTP C (50%).	LUMItest PCT (B.R.A.H.M.S.- Diagnostica, Berlin, Germany)	Simultaneously with ascitic fluid samples
Viallon	2000	French	NA	ED	61	34.4%	58.0	PCT, CRP,	Cirrhosis	Ascites PMN ≥	NA	LUMItest PCT	Before

		IL-6, TNF-α, PMN	250/mm³	(B.R.A.H.M.S.-Diagnostica, Berlin, Germany)	antibiotics

SBP: spontaneous bacterial peritonitis; PCT: procalcitonin; CRP: C-reactive protein; PMN: polymorphonuclear neutrophil; CTP: Child-Turcotte-Pugh; NA: not available; PS: prospective cohort study; RS: retrospective cohort study; WBC: white blood cell; PLT: platelet; sNFI: mean fluorescence intensity of mature neutrophils; dCHC: difference in hemoglobin concentration between newly formed and mature red blood cells; TNF-α: tumor necrosis factor-alpha; IL-6: interleukin-6; HCC: hepatocellular carcinoma; MIP-1β: macrophage inflammatory protein-1 beta; HNP: human neutrophil peptide

[†] When the mean and standard deviation of the age of the whole group were not reported, we calculated these values by combining two groups sequentially using Cochrane's formula.

Supplementary Table S4. Risk of bias and concerns of applicability for the 20 studies included in this review

Study	Patient Selection	Index test	Reference Standard	Flow and timing
Datta 2023	Risk of Bias: high<br>• Convenience sampling<br>• Retrospective cohort cohort<br>• Avoided inappropriate exclusion<br><br>Concerns about applicability: low<br>• Appropriate study population	Risk of Bias: high<br>• Optimal cutoff<br>Concerns about applicability: low<br>• Index test conduct and execution clear<br>• Index test interpretation clear	Risk of Bias: low<br>• Same outcomes assessed for all biomarkers<br>• Outcome assessment independent of test<br>• Adequate descriptions of reference standards<br>Concerns about applicability: low<br>• Relevant outcomes	Risk of Bias: low<br>• All patients received test<br>• All patient outcomes assessed<br>• Appropriate interval between index test and reference standard
Xiang 2022	Risk of Bias: low<br>• Selection process clear<br>• Retrospective cohort study	Risk of Bias: low<br>• Predefined cutoff<br><br>Concerns about applicability: low	Risk of Bias: low<br>• Same outcomes assessed for all biomarkers<br>• Outcome assessment independent of test	Risk of Bias: unclear<br>• All patients received test<br>• All patient outcomes assessed

	• Avoided inappropriate exclusion<br>Concerns about applicability: low<br>• Appropriate study population	• Index test conduct and execution clear	• Adequate descriptions of reference standards<br>Concerns about applicability: low<br>• Relevant outcomes	• Time of blood draw unknown
Verma 2022	Risk of Bias: low<br>• Selection process clear<br>• Prospective cohort study<br>• Avoided inappropriate exclusion<br>Concerns about applicability: low<br>• Appropriate study population	Risk of Bias: low<br>• Predefined cutoff<br>Concerns about applicability: low<br>• Index test, conduct and interpretation clear	Risk of Bias: low<br>• Same outcomes assessed for all biomarkers<br>• Outcome assessment independent of test<br>• Adequate descriptions of reference standards<br>Concerns about applicability: low<br>• Relevant outcomes	Risk of Bias: low<br>• All patients received test<br>• All patient outcomes assessed<br>• Appropriate interval between index test and reference standard
Mohamed 2022	Risk of Bias: low	Risk of Bias: high<br>• Optimal cutoff	Risk of Bias: low	Risk of Bias: unclear

	• Selection process clear<br>• Cohort study<br>• Avoided inappropriate exclusion<br>Concerns about applicability: high<br>• Appropriate study population	Concerns about applicability: low<br>• Index test conduct and execution clear	• Same outcomes assessed for all biomarkers<br>• Outcome assessment independent of test<br>• Adequate descriptions of reference standards<br>Concerns about applicability: low<br>• Relevant outcomes	• All patients received test<br>• All patient outcomes assessed<br>• Time of blood draw unknown
Jiang 2021	Risk of Bias: low<br>• Selection process clear<br>• Prospective cohort study<br>• Avoided inappropriate exclusion<br>Concerns about applicability: low	Risk of Bias: high<br>• Optimal cutoff<br>Concerns about applicability: low<br>• Index test conduct and execution clear	Risk of Bias: low<br>• Same outcomes assessed for all biomarkers<br>• Outcome assessment independent of test<br>• Adequate descriptions of reference standards<br>Concerns about applicability: low<br>• Relevant outcomes	Risk of Bias: low<br>• All patients received test<br>• All patient outcomes assessed<br>• Appropriate interval between index test and reference standard

	• Appropriate study population			
El-Hassib 2021	Risk of Bias: low<br>• Selection process clear<br>• Prospective cohort study<br>• Avoided inappropriate exclusion<br><br>Concerns about applicability: low<br>• Appropriate study population	Risk of Bias: high<br>• Optimal cutoff<br><br>Concerns about applicability: low<br>• Index test conduct and execution clear	Risk of Bias: low<br>• Same outcomes assessed for all biomarkers<br>• Outcome assessment independent of test<br>• Adequate descriptions of reference standards<br>Concerns about applicability: low<br>• Relevant outcomes	Risk of Bias: low<br>• All patients received test<br>• All patient outcomes assessed<br>• Appropriate interval between index test and reference standard
Mohamed 2021	Risk of Bias: low<br>• Selection process clear<br>• Prospective cohort study	Risk of Bias: high<br>• Optimal cutoff<br><br>Concerns about applicability: low<br>Index test conduct and execution clear	Risk of Bias: low<br>• Same outcomes assessed for all biomarkers<br>• Outcome assessment independent of test	Risk of Bias: unclear<br>• All patients received test<br>• All patient outcomes assessed<br>• Unknown time of blood draw

	• Avoided inappropriate exclusion<br><br>Concerns about applicability: unclear<br>• Include only HCV cirrhotic patients		• Adequate descriptions of reference standards<br>Concerns about applicability: low<br>Relevant outcomes	
Wang 2018	Risk of Bias: low<br>• Selection process clear<br>• Prospective cohort study<br>• Exclusion criteria clear and appropriate<br><br>Concerns about applicability: low<br>• Appropriate study population	Risk of Bias: high<br>• Optimal cutoff<br><br>Concerns about applicability: low<br>• Index test conduct and execution clear	Risk of Bias: low<br>• Same outcome assessed for all biomarkers<br>• Outcome assessment independent of test<br>• Adequate descriptions of reference standards<br><br>Concerns about applicability: low<br>Relevant outcomes	Risk of Bias: low<br>• All patients received test<br>• All patient outcomes assessed<br>• interval between index test and reference standard

Mikuła 2018	Risk of Bias: low<br>• Selection process clear<br>• Prospective study<br>• Exclusion criteria clear and appropriate<br>Concerns about applicability: low<br>• Appropriate study population	Risk of Bias: low<br>• Predefined cutoff<br>Concerns about applicability: low<br>• Index test conduct and execution clear	Risk of Bias: low<br>• Same outcomes assessed for all biomarkers<br>• Outcome assessment independent of test<br>• Adequate descriptions of reference standards<br>Concerns about applicability: low<br>• Relevant outcomes	Risk of Bias: low<br>• Not all patients received tests and were included in the analysis<br>• All patient outcomes assessed<br>• Appropriate interval between index test and reference standard
Şahintürk 2016	Risk of Bias: low<br>• Selection process clear<br>• Prospective cohort study<br>• Exclusion criteria clear and appropriate<br>Concerns about applicability: low	Risk of Bias: low<br>• Predfined cutoff<br>Concerns about applicability: low<br>• Index test conduct and execution clear	Risk of Bias: low<br>• Same outcome assessed for all biomarkers<br>• Outcome assessment independent of test<br>• Adequate descriptions of reference standards<br>Concerns about applicability: low	Risk of Bias: low<br>• All patients received test<br>• All patient outcomes assessed<br>• Appropriate interval between index test and reference standard

	• Appropriate study population		• Relevant outcomes	
Li 2016	Risk of Bias: low<br>• Selection process clear<br>• Retrospective cohort study<br>• Exclusion criteria clear and appropriate<br><br>Concerns about applicability: low<br>• Appropriate study population	Risk of Bias: high<br>• Optimal cutoff<br><br>Concerns about applicability: low<br>• Index test conduct and execution clear	Risk of Bias: low<br>• Same outcomes assessed for all biomarkers<br>• Outcome assessment independent of test<br>• Adequate descriptions of reference standards<br>Concerns about applicability: low<br>• Relevant outcomes	Risk of Bias: low<br>• All patients received test<br>• All patient outcomes assessed<br>• Appropriate interval between index test and reference standard
Abdel-Razik 2016	Risk of Bias: low<br>• Selection process clear<br>• Prospective cohort study	Risk of Bias: high<br>• Optimal cutoff<br><br>Concerns about applicability: low	Risk of Bias: low<br>• Same outcomes assessed for all biomarkers<br>• Outcome assessment independent of test	Risk of Bias: low<br>• All patients received test<br>• All patient outcomes assessed

	• Exclusion criteria clear and appropriate<br>Concerns about applicability: unclear<br>• Include only cirrhotic patients with HCC for ablation therapy	• Index test conduct and execution clear	• Adequate descriptions of reference standards<br>Concerns about applicability: low<br>• Relevant outcomes	• Appropriate interval between index test and reference standard
Cai 2015	Risk of Bias: high<br>• Selection process clear<br>• Retrospective case-control study<br>• Exclusion criteria clear and appropriate<br>Concerns about applicability: low<br>• Appropriate study population	Risk of Bias: low<br>• Predefined cutoff<br>Concerns about applicability: low<br>• Index test conduct and execution clear	Risk of Bias: low<br>• Same outcomes assessed for all biomarkers<br>• Outcome assessment independent of test<br>• Adequate descriptions of reference standards<br>Concerns about applicability: low<br>• Relevant outcomes	Risk of Bias: low<br>• All patients received test<br>• All patient outcomes assessed<br>• Appropriate interval between index test and reference standard
Wu 2014	Risk of Bias: low	Risk of Bias: high<br>• Optimal cutoff	Risk of Bias: low	Risk of Bias: low

	• Selection process clear<br>• Retrospective cohort study<br>• Exclusion criteria clear and appropriate<br><br>Concerns about applicability: low<br>• Appropriate study population	Concerns about applicability: low<br>• Index test conduct and execution clear	• Same outcomes assessed for all biomarkers<br>• Outcome assessment independent of test<br>• Adequate descriptions of reference standards<br>Concerns about applicability: low<br>• Relevant outcomes	• All patients received test<br>• All patient outcomes assessed<br>• Appropriate interval between index test and reference standard
Lesińska 2014	Risk of Bias: low<br>• Selection process clear<br>• Prospective cohort study<br>• Exclusion criteria clear and appropriate<br><br>Concerns about applicability: low<br>• Appropriate study population	Risk of Bias: low<br>• Predefined cutoff<br><br>Concerns about applicability: low<br>• Index test conduct and execution clear	Risk of Bias: low<br>• Same outcomes assessed for all biomarkers<br>• Outcome assessment independent of test<br>• Adequate descriptions of reference standards<br>Concerns about applicability: low<br>• Relevant outcomes	Risk of Bias: low<br>• All patients received test<br>• All patient outcomes assessed<br>• Appropriate interval between index test and reference standard

Yuan 2013	Risk of Bias: low<br>• Selection process clear<br>• Retrospective cohort study<br>• Exclusion criteria clear and appropriate<br><br>Concerns about applicability: unclear<br>• Include only chronic severe hepatitis B	Risk of Bias: high<br>• Optimal cutoff<br><br>Concerns about applicability: low<br>• Index test conduct and execution clear	Risk of Bias: low<br>• Same outcomes assessed for all biomarkers<br>• Outcome assessment independent of test<br>• Adequate descriptions of reference standards<br>Concerns about applicability: low<br>• Relevant outcomes	Risk of Bias: low<br>• All patients received test<br>• All patient outcomes assessed<br>• Appropriate interval between index test and reference standard
Gong 2013	Risk of Bias: low<br>• Selection process clear<br>• Retrospective cohort study<br>• Exclusion criteria clear and appropriate	Risk of Bias: low<br>• Predefined cutoff<br><br>Concerns about applicability: low<br>• Index test conduct and execution clear	Risk of Bias: low<br>• Same outcome assessed for all biomarkers<br>• Outcome assessment independent of test	Risk of Bias: low<br>• All patients received test<br>• All patient outcomes assessed<br>• Appropriate interval between index test

	Concerns about applicability: low<br>• Appropriate study population		• Adequate descriptions of reference standards<br>Concerns about applicability: low<br>• Relevant outcomes	and reference standard
Cekin 2013	Risk of Bias: low<br>• Selection process clear<br>• Retrospective cohort study<br>• Exclusion criteria clear and appropriate<br><br>Concerns about applicability: unclear<br>• Include cirrhosis-related or malignancy-related ascites	Risk of Bias: high<br>• Optimal cutoff<br><br>Concerns about applicability: low<br>• Index test conduct and execution clear	Risk of Bias: low<br>• Same outcome assessed for all biomarkers<br>• Outcome assessment independent of test<br>• Adequate descriptions of reference standards<br>Concerns about applicability: low<br>• Relevant outcomes	Risk of Bias: low<br>• All patients received test<br>• All patient outcomes assessed<br>• Appropriate interval between index test and reference standard
Spahr 2001	Risk of Bias: low	Risk of Bias: low	Risk of Bias: low	Risk of Bias: low

	• Selection process clear<br>• Cohort study<br>• Exclusion criteria clear and appropriate<br>Concerns about applicability: low<br>• Appropriate study population	• Predefined cutoff<br><br>Concerns about applicability: low<br>• Index test conduct and execution clear	• Same outcomes assessed for all biomarkers<br>• Outcome assessment independent of test<br>• Adequate descriptions of reference standards<br>Concerns about applicability: low<br>• Relevant outcomes	• All patients received test<br>• All patient outcomes assessed<br>• Appropriate interval between index test and reference standard
Viallon 2000	Risk of Bias: low<br>• Selection process clear<br>• Cohort study<br>• Exclusion criteria clear and appropriate<br><br>Concerns about applicability: low<br>• Appropriate study population	Risk of Bias: high<br>• Optimal cutoff<br><br>Concerns about applicability: low<br>• Index test conduct and execution clear	Risk of Bias: low<br>• Same outcome assessed for all biomarkers<br>• Outcome assessment independent of test<br>• Adequate descriptions of reference standards<br>Concerns about applicability: low<br>• Relevant outcomes	Risk of Bias: low<br>• All patients received test<br>• All patient outcomes assessed<br>• Appropriate interval between index test and reference standard

Supplementary Table S5. GRADE evidence profile to determine the diagnostic accuracy of procalcitonin and C-reactive protein

**PCT**	Sensitivity 0.73 (95% CI, 0.61-0.83); Specificity 0.88 (95% CI, 0.83-0.91)										
Outcome	Number of studies (patients)	Study design	Factors that my decrease certainty of evidence					Effect per 1,000 patients tested			Test accuracy CoE
			Risk of bias	Indirectness	Inconsistency	Imprecision	Publication bias	Pretest probability of 30%	Pretest probability of 50%	Pretest probability of 70%	
True positive	20 (6,044)	Observational	Serious[a]	Not serious	Serious[b]	Not serious	Serious	219 (183,249)	365 (305,415)	511 (427,581)	⊕○○○ Very low
False negative								81 (51,117)	135 (85,195)	189 (119,273)	
True negative	20 (6,044)	Observational	Serious[a]	Not serious	Not serious[b]	Not serious	Serious	616 (581,637)	440 (415,455)	264 (249,273)	⊕⊕○○ Low
False positive								84 (63,119)	60 (45,85)	36 (27,51)	

**CRP**	Sensitivity 0.70 (95% CI, 0.59-0.79); Specificity 0.73 (95% CI, 0.60-0.82)										
Outcome	Number of studies (patients)	Study design	Factors that my decrease certainty of evidence					Effect per 1000 patients tested			Test accuracy CoE
			Risk of bias	Indirectness	Inconsistency	Imprecision	Publication bias	Pretest probability of 40%	Pretest probability of 55%	Pretest probability of 70%	

True positive<br>False negative	10 (4,840)	Observational	Serious[c]	Not serious	Not serious[d]	Not serious	Not serious	210 (177,237)<br>90 (63,123)	350 (295,395)<br>150 (105,205)	490 (413,553)<br>210 (147,287)	⊕⊕⊕○ Moderate
True negative<br>False positive	10 (4,840)	Observational	Serious[c]	Not serious	Not serious[d]	Not serious	Not serious	511 (420,574)<br>189 (126,280)	365 (300,410)<br>135 (90,200)	219 (180,246)<br>81 (54,120)	⊕⊕⊕○ Moderate

[a] As assessed by QUADAS-2, within the patient selection domain, two studies (10%) were deemed to carry a high risk of bias due to one of case-control design and one of non-consecutive patient enrollment. In the domain of the index test, 13 (65%) studies were associated with a high risk of bias since they calculated sensitivity and specificity using the optimal cutoff value other than the predefined value. In the flow and timing domain, three studies (15%) exhibited an unclear risk of bias because the time of blood sample collection is not documented. Regarding applicability, four studies (20%) displayed an unclear risk of bias in the patient selection domain as they enrolled specific population. We downgraded one level.

[b] For individual studies, sensitivity estimates ranged from 0.19 to 1, and specificity estimates ranged from 0.72 to 1. We thought that even cutoffs could not explain the heterogeneity of sensitivity. Therefore, we downgraded the sensitivity by one level.

[c] As assessed by QUADAS-2, within the patient selection domain, one studies (10%) were deemed to carry a high risk of bias due to non-consecutive patient enrollment. In the domain of the index test, six (60%) studies were associated with a high risk of bias since they calculated sensitivity and specificity using the optimal cutoff value other than the predefined value. In the flow and timing domain, two studies (20%) exhibited an unclear risk of bias because the time of blood sample collection is not documented. Regarding applicability, one studies (10%) displayed an unclear risk of bias in the patient selection domain as they enrolled specific population. We downgraded one level.

[d] For individual studies, sensitivity estimates ranged from 0.52 to 1, and specificity estimates ranged from 0.06 to 0.95. We thought that the

cutoffs could explain some of the heterogeneity. We did not downgrade the evidence.

PCT: procalcitonin; CRP: C-reactive protein; CoE: certainty of evidence; QUADAS-2: Quality Assessment of Diagnostic Accuracy Studies-2
GRADE Certainty of the evidence
High: we are very confident that the true effect lies close to that of the estimate of the effect. Moderate: we are moderately confident in the effect estimate: the true effect is likely to be close to the estimate of the effect, but there is a possibility that it is substantially different. Low: our confidence in the effect estimate is limited: the true effect may be substantially different from the estimate of the effect. Very low: we have very little confidence in the effect estimate: the true effect is likely to be substantially different from the estimate of effect.

Supplementary Figure S1. Quality assessment for 44 studies (QUADAS-2)

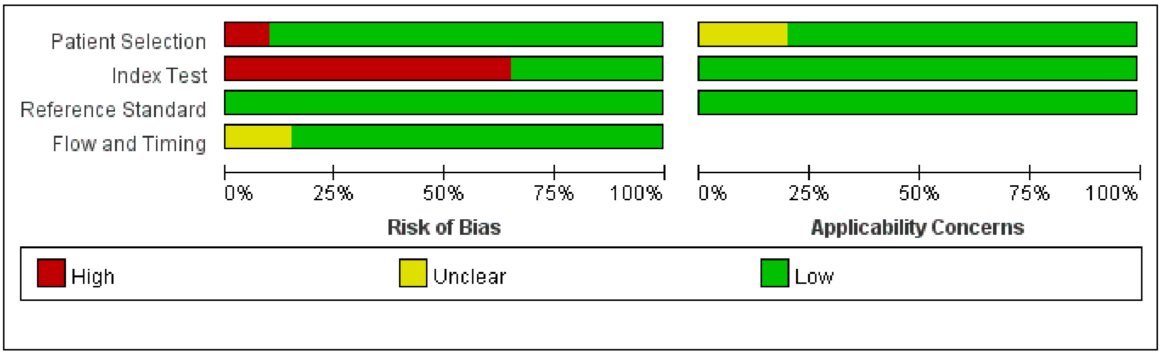

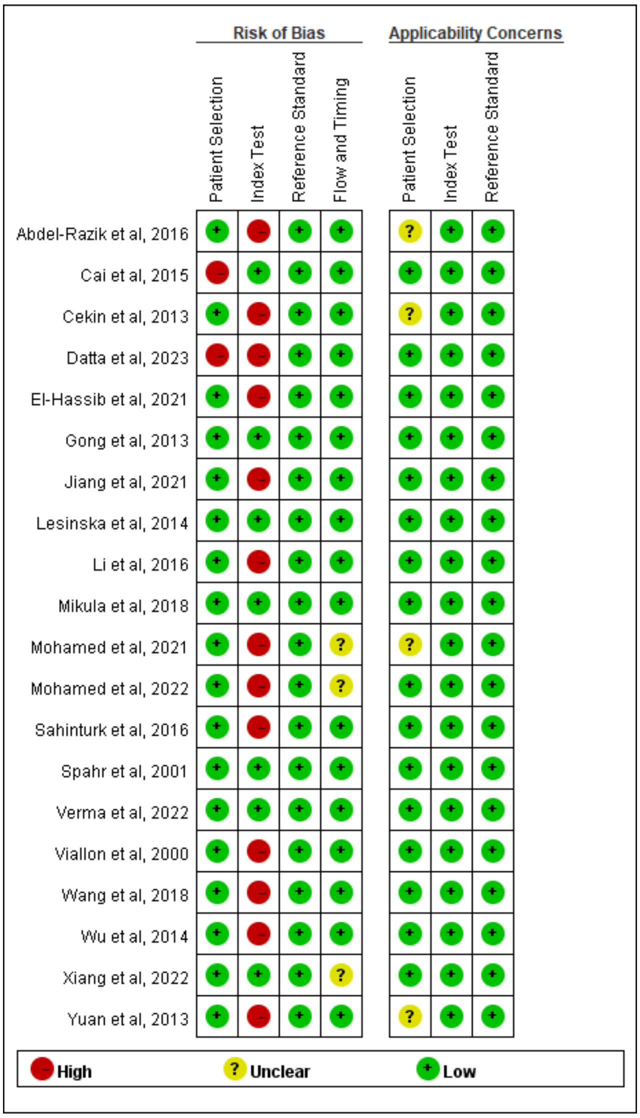

Supplementary Figure S2. (A) SROC curves and (B) forest plots for the diagnostic accuracy of procalcitonin for spontaneous bacterial peritonitis in studies using a cutoff of 0.5-1 ng/mL. SROC: summary receiver operating characteristic; TP: true positive; FP: false positive; FN: false negative; TN: true negative; CI: confidence interval.

(A)

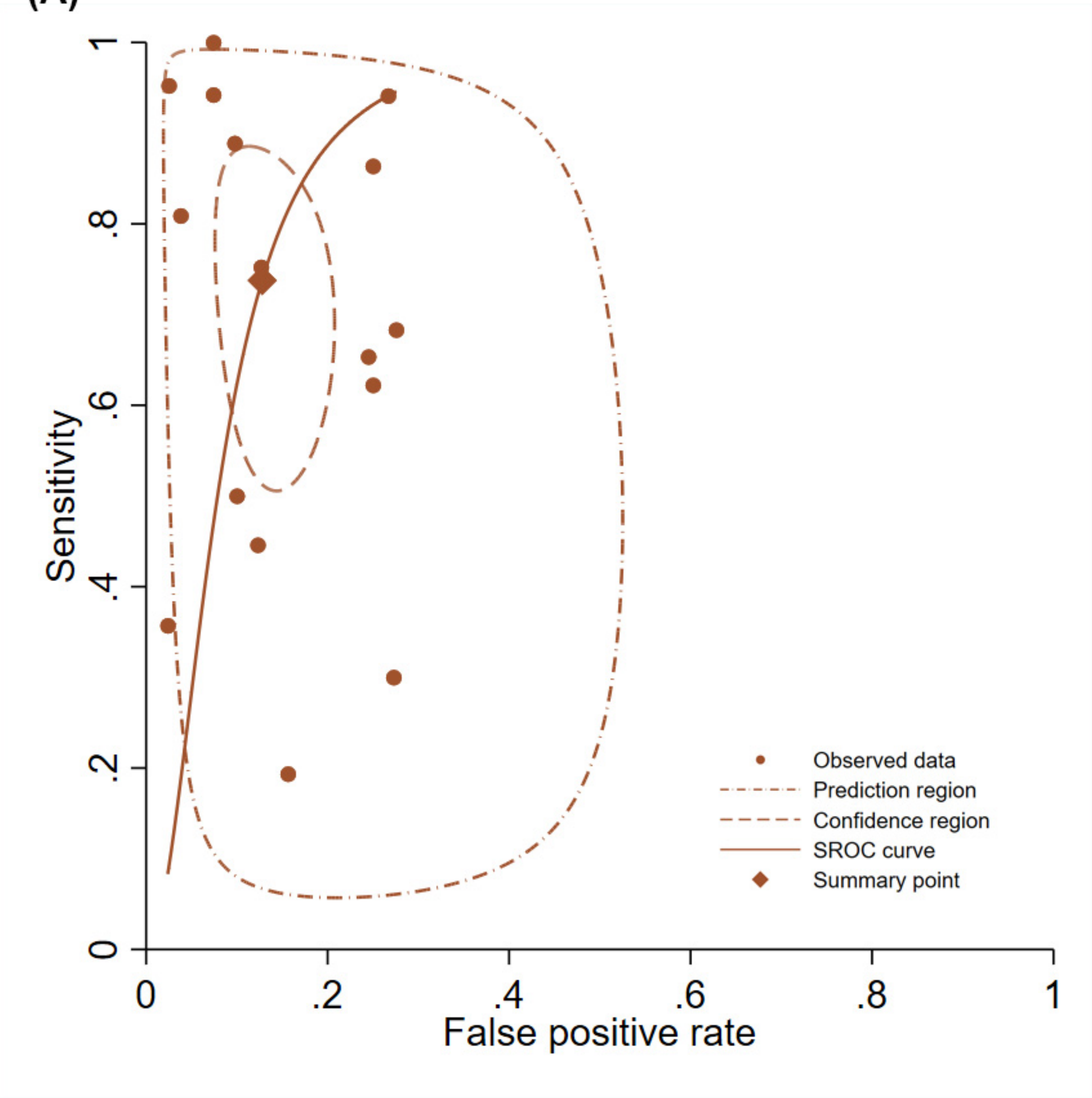

(B)

Study	TP	FP	FN	TN	Cutoff (ng/mL)	Sensitivity (95% CI)	Specificity (95% CI)
Datta et al, 2023	8	4	1	37	0.67	0.89 [0.52, 1.00]	0.90 [0.77, 0.97]
Xiang et al, 2022	355	374	441	2667	0.5	0.45 [0.41, 0.48]	0.88 [0.86, 0.89]
Mohamed et al, 2022	66	24	35	74	1.0	0.65 [0.55, 0.75]	0.76 [0.66, 0.84]
El-Hassib et al, 2021	69	27	32	71	0.95	0.68 [0.58, 0.77]	0.72 [0.63, 0.81]
Mohamed et al, 2021	16	8	1	22	0.7	0.94 [0.71, 1.00]	0.73 [0.54, 0.88]
Wang et al, 2018	82	19	27	131	0.88	0.75 [0.66, 0.83]	0.87 [0.81, 0.92]
Mikula et al, 2018	6	5	25	27	0.5	0.19 [0.07, 0.37]	0.84 [0.67, 0.95]
Sahinturk et al, 2016	19	7	3	21	0.5	0.86 [0.65, 0.97]	0.75 [0.55, 0.89]
Abdel-Razik et al, 2016	49	2	3	25	0.94	0.94 [0.84, 0.99]	0.93 [0.76, 0.99]
Wu et al, 2014	144	7	34	177	0.5	0.81 [0.74, 0.86]	0.96 [0.92, 0.98]
Lesinska et al, 2014	3	6	7	16	0.5	0.30 [0.07, 0.65]	0.73 [0.50, 0.89]
Yuan et al, 2013	15	1	27	41	0.67	0.36 [0.22, 0.52]	0.98 [0.87, 1.00]
Gong et al, 2013	28	8	17	24	0.5	0.62 [0.47, 0.76]	0.75 [0.57, 0.89]
Cekin et al, 2013	20	6	0	75	0.61	1.00 [0.83, 1.00]	0.93 [0.85, 0.97]
Spahr et al, 2001	5	1	5	9	0.5	0.50 [0.19, 0.81]	0.90 [0.55, 1.00]
Viallon et al, 2000	20	1	1	39	0.76	0.95 [0.76, 1.00]	0.97 [0.87, 1.00]

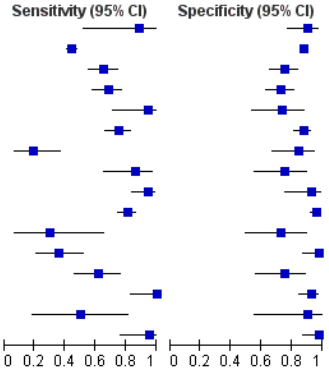

Supplementary Figure S3. (A) SROC curves and (B) forest plots for the diagnostic accuracy of procalcitonin for spontaneous bacterial peritonitis in studies including 100% cirrhotic patients. SROC: summary receiver operating characteristic; TP: true positive; FP: false positive; FN: false negative; TN: true negative; CI: confidence interval.

(A)

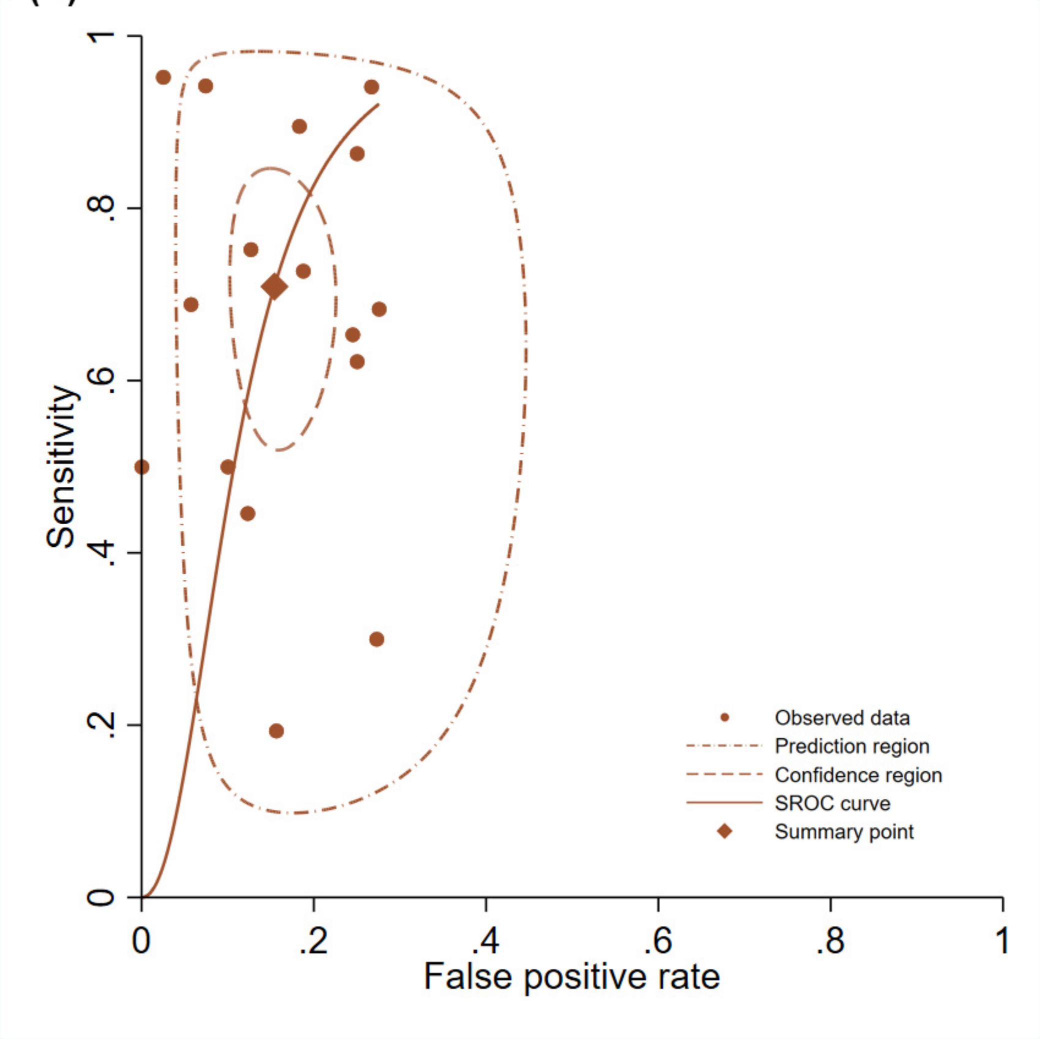

(B)

Study	TP	FP	FN	TN	Cutoff (ng/mL)	Sensitivity (95% CI)	Specificity (95% CI)
Mohamed et al, 2022	66	24	35	74	1.0	0.65 [0.55, 0.75]	0.76 [0.66, 0.84]
Verma et al, 2022	7	0	7	31	2.0	0.50 [0.23, 0.77]	1.00 [0.89, 1.00]
Xiang et al, 2022	355	374	441	2667	0.5	0.45 [0.41, 0.48]	0.88 [0.86, 0.89]
El-Hassib et al, 2021	69	27	32	71	0.95	0.68 [0.58, 0.77]	0.72 [0.63, 0.81]
Jiang et al, 2021	40	30	15	130	0.35	0.73 [0.59, 0.84]	0.81 [0.74, 0.87]
Mohamed et al, 2021	16	8	1	22	0.7	0.94 [0.71, 1.00]	0.73 [0.54, 0.88]
Wang et al, 2018	82	19	27	131	0.88	0.75 [0.66, 0.83]	0.87 [0.81, 0.92]
Mikula et al, 2018	6	5	25	27	0.5	0.19 [0.07, 0.37]	0.84 [0.67, 0.95]
Li et al, 2016	77	15	9	67	1.9	0.90 [0.81, 0.95]	0.82 [0.72, 0.89]
Sahinturk et al, 2016	19	7	3	21	0.5	0.86 [0.65, 0.97]	0.75 [0.55, 0.89]
Abdel-Razik et al, 2016	49	2	3	25	0.94	0.94 [0.84, 0.99]	0.93 [0.76, 0.99]
Cai et al, 2015	42	2	19	33	2.0	0.69 [0.56, 0.80]	0.94 [0.81, 0.99]
Lesinska et al, 2014	3	6	7	16	0.5	0.30 [0.07, 0.65]	0.73 [0.50, 0.89]
Gong et al, 2013	28	8	17	24	0.5	0.62 [0.47, 0.76]	0.75 [0.57, 0.89]
Spahr et al, 2001	5	1	5	9	0.5	0.50 [0.19, 0.81]	0.90 [0.55, 1.00]
Viallon et al, 2000	20	1	1	39	0.76	0.95 [0.76, 1.00]	0.97 [0.87, 1.00]

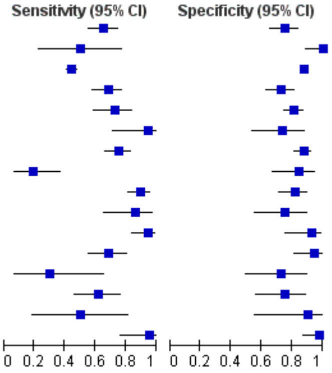

Supplementary Figure S4. (A) SROC curves and (B) forest plots for the diagnostic accuracy of procalcitonin for spontaneous bacterial peritonitis in studies excluding case-control design. SROC: summary receiver operating characteristic; TP: true positive; FP: false positive; FN: false negative; TN: true negative; CI: confidence interval.

(A)

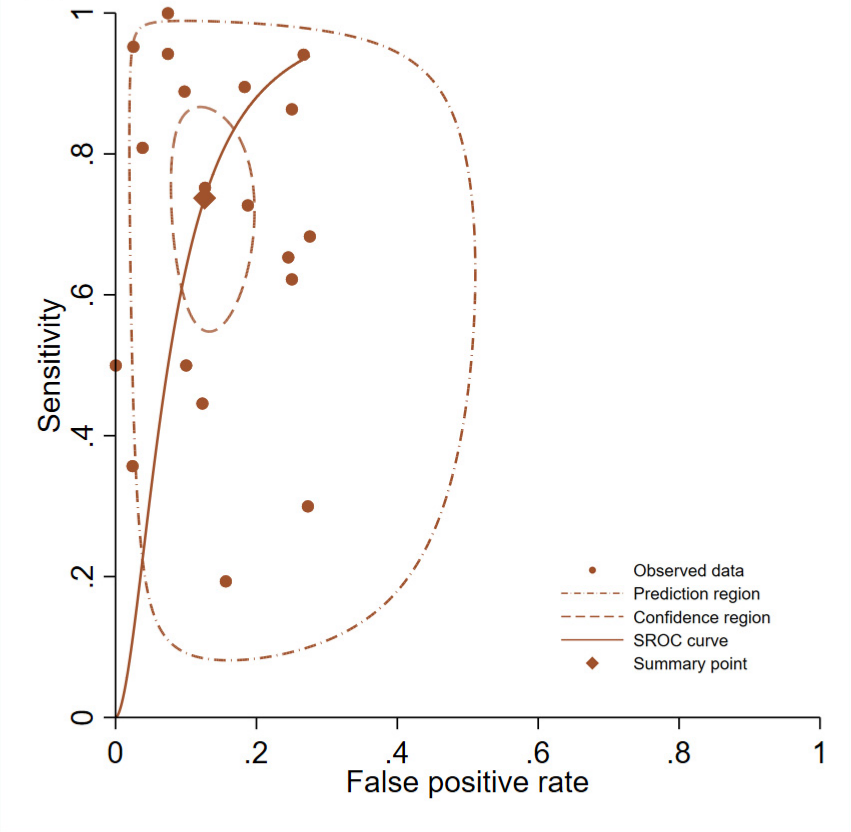

(B)

Study	TP	FP	FN	TN	Cutoff (ng/mL)	Sensitivity (95% CI)	Specificity (95% CI)
Mohamed et al, 2022	66	24	35	74	1.0	0.65 [0.55, 0.75]	0.76 [0.66, 0.84]
Verma et al, 2022	7	0	7	31	2.0	0.50 [0.23, 0.77]	1.00 [0.89, 1.00]
Xiang et al, 2022	355	374	441	2667	0.5	0.45 [0.41, 0.48]	0.88 [0.86, 0.89]
El-Hassib et al, 2021	69	27	32	71	0.95	0.68 [0.58, 0.77]	0.72 [0.63, 0.81]
Jiang et al, 2021	40	30	15	130	0.35	0.73 [0.59, 0.84]	0.81 [0.74, 0.87]
Mohamed et al, 2021	16	8	1	22	0.7	0.94 [0.71, 1.00]	0.73 [0.54, 0.88]
Wang et al, 2018	82	19	27	131	0.88	0.75 [0.66, 0.83]	0.87 [0.81, 0.92]
Mikula et al, 2018	6	5	25	27	0.5	0.19 [0.07, 0.37]	0.84 [0.67, 0.95]
Li et al, 2016	77	15	9	67	1.9	0.90 [0.81, 0.95]	0.82 [0.72, 0.89]
Sahinturk et al, 2016	19	7	3	21	0.5	0.86 [0.65, 0.97]	0.75 [0.55, 0.89]
Abdel-Razik et al, 2016	49	2	3	25	0.94	0.94 [0.84, 0.99]	0.93 [0.76, 0.99]
Cai et al, 2015	42	2	19	33	2.0	0.69 [0.56, 0.80]	0.94 [0.81, 0.99]
Lesinska et al, 2014	3	6	7	16	0.5	0.30 [0.07, 0.65]	0.73 [0.50, 0.89]
Gong et al, 2013	28	8	17	24	0.5	0.62 [0.47, 0.76]	0.75 [0.57, 0.89]
Spahr et al, 2001	5	1	5	9	0.5	0.50 [0.19, 0.81]	0.90 [0.55, 1.00]
Viallon et al, 2000	20	1	1	39	0.76	0.95 [0.76, 1.00]	0.97 [0.87, 1.00]

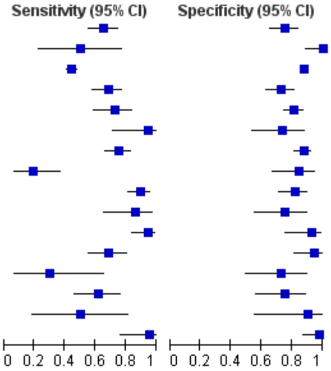

Supplementary Figure S5. (A) SROC curves and (B) forest plots for the diagnostic accuracy of procalcitonin for spontaneous bacterial peritonitis in studies excluding those using optimal cutoffs. SROC: summary receiver operating characteristic; TP: true positive; FP: false positive; FN: false negative; TN: true negative; CI: confidence interval.

(A)

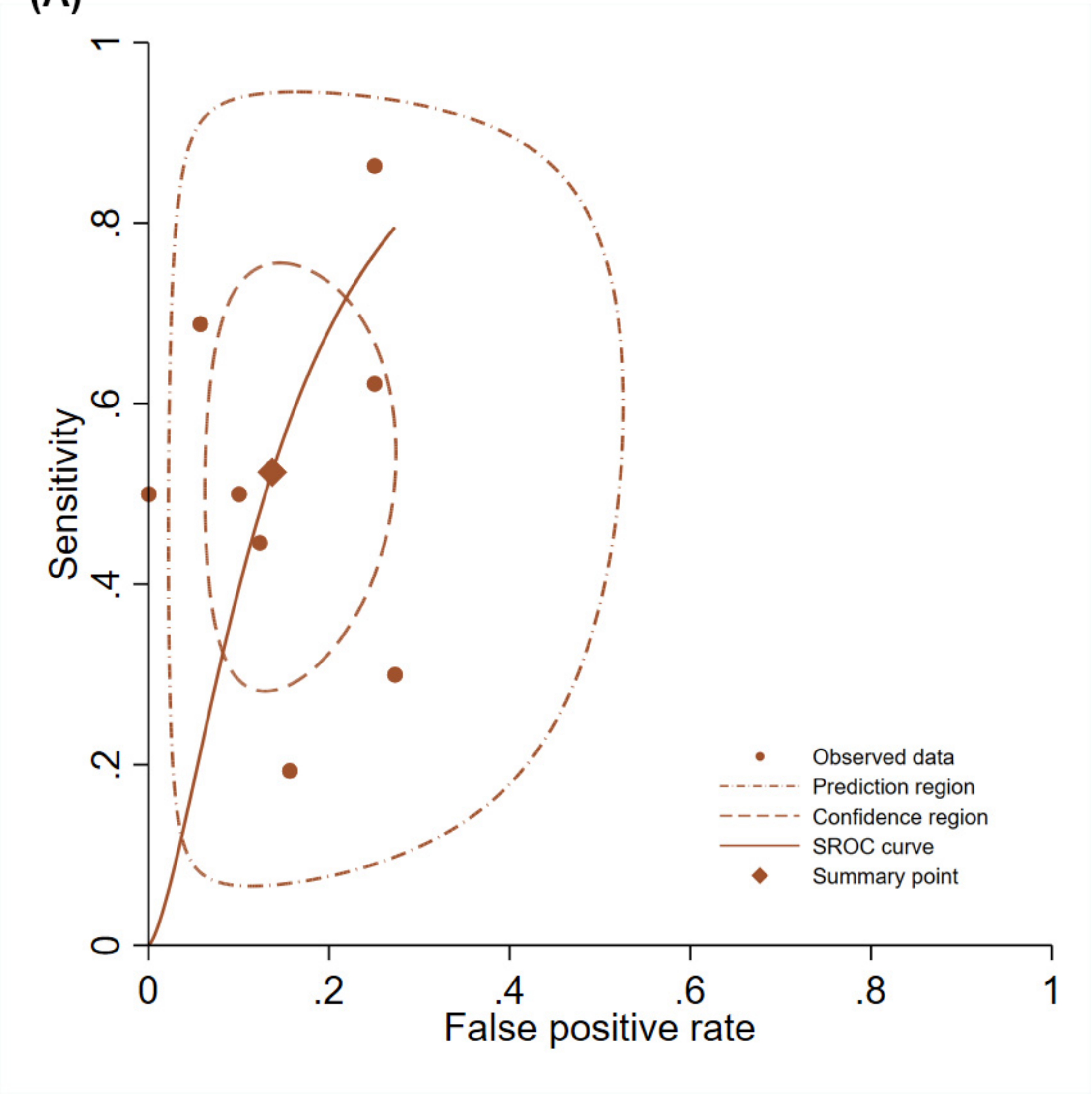

(B)

Study	TP	FP	FN	TN	Cutoff (ng/mL)	Sensitivity (95% CI)	Specificity (95% CI)
Xiang et al, 2022	355	374	441	2667	0.5	0.45 [0.41, 0.48]	0.88 [0.86, 0.89]
Verma et al, 2022	7	0	7	31	2.0	0.50 [0.23, 0.77]	1.00 [0.89, 1.00]
Mikula et al, 2018	6	5	25	27	0.5	0.19 [0.07, 0.37]	0.84 [0.67, 0.95]
Sahinturk et al, 2016	19	7	3	21	0.5	0.86 [0.65, 0.97]	0.75 [0.55, 0.89]
Abdel-Razik et al, 2016	49	2	3	25	0.94	0.94 [0.84, 0.99]	0.93 [0.76, 0.99]
Cai et al, 2015	42	2	19	33	2.0	0.69 [0.56, 0.80]	0.94 [0.81, 0.99]
Lesinska et al, 2014	3	6	7	16	0.5	0.30 [0.07, 0.65]	0.73 [0.50, 0.89]
Gong et al, 2013	28	8	17	24	0.5	0.62 [0.47, 0.76]	0.75 [0.57, 0.89]
Spahr et al, 2001	5	1	5	9	0.5	0.50 [0.19, 0.81]	0.90 [0.55, 1.00]

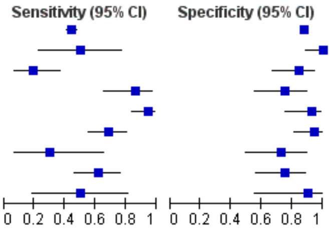

Supplementary Figure S6. Deeks' funnel plot (asymmetry test) for (A) PCT and (B) CRP. PCT: procalcitonin; CRP: C-reactive protein.

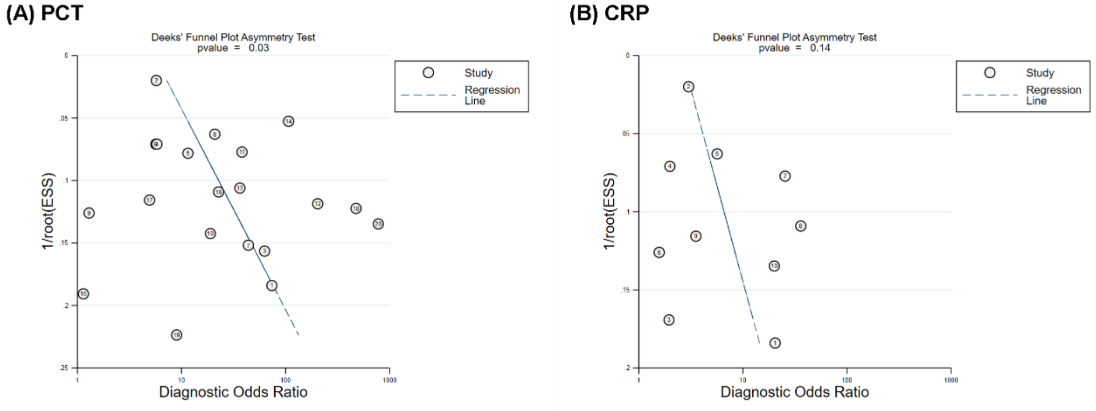

Supplement: Supplementary file 1 [file medicina-61-01134-s001.zip › medicina-3666012-supplementary.pdf]
